# Remote Ischemic Postconditioning Protects against Myocardial Ischemia-Reperfusion Injury by Inhibition of the RAGE-HMGB1 Pathway

**DOI:** 10.1155/2018/4565630

**Published:** 2018-01-23

**Authors:** Xiangming Wang, Junhong Wang, Tiantian Tu, Zakaria Iyan, Deeraj Mungun, Zhijian Yang, Yan Guo

**Affiliations:** ^1^Department of Gerontology, The First Affiliated Hospital of Nanjing Medical University, Nanjing, China; ^2^Department of Cardiology, The First Affiliated Hospital of Nanjing Medical University, Nanjing, China; ^3^Department of Cardiology, Jiangsu Shengze Hospital, Suzhou, China

## Abstract

**Background:**

The aim of the present study was to observe the effect of RAGE-HMGB1 signal pathway on remote ischemic postconditioning in mice with myocardial ischemia reperfusion injury.

**Methods:**

Mice model of MIRI was established and randomly divided into three groups: control group, ischemia reperfusion group, and remote ischemic postconditioning group. Infarction size was detected by Evans blue and TTC staining. Cardiac function was detected by echocardiography measurement. The protein levels of RAGE, HMGB1, P-AKT, and ERK1/2 were detected by Western blot 120 min following reperfusion.

**Results:**

RIPostC could decrease the infarct size and increase LVEF and FS compared with I/R group. Two hours after myocardial ischemia reperfusion, the levels of RAGE and HMGB1 were significantly decreased in RIPostC group compared with those in I/R group. The level of p-AKT was significantly higher in the RIPostC group than in the I/R group. LY294002 significantly attenuated RIPostC-increased levels of Akt phosphorylation.

**Conclusion:**

RIPostC may inhibit the expression of RAGE and HMGB1 and activate PI3K/Akt signaling pathway to extenuate ischemic reperfusion injury in mice. It could further suppress the oxidative stress, have antiapoptosis effect, and reduce inflammatory reaction, but this effect has certain timeliness.

## 1. Introduction

In acute myocardial infarction, timely and effective myocardial reperfusion therapy, such as PCI, CABG, and thrombolytic, is the effective method to limit myocardial infarct area, attenuate clinical symptoms, and improve clinical prognosis. However, the beneficial effects can be compromised by ischemia/reperfusion injury [1, 2]. Myocardial ischemia-reperfusion injury is a common pathophysiological process, a serious threat to the health of patients. I/R can induce further damage to the myocardium itself, leading to cell dysfunction and death [3, 4]. The main mechanism and developing process of ischemia-reperfusion injury may occur with oxidative stress and inflammatory reaction.

Remote ischemic conditioning (RIC), induced by several episodes of brief ischemia and reperfusion at a distance organ, has to be developed as an effective strategy of interorgan protection against the harmful effects of acute ischemia-reperfusion injury in such a way that it has been extended to different tissues and organs. Not only RIPC has a protective effect on the heart, but also the concept of RIPC has been further extended to protect multiple organs such as kidney, liver, and brain. RIC has been shown to reduce myocardial ischemia-reperfusion injury in several animal models. Remote ischemic postconditioning (RIPostC) has been verified to have beneficial effect in animal studies [5, 6] and randomized clinical trials [7, 8]. However, the underlying mechanism of RIPostC is still poorly understood. The mechanism of RIPostC is very complex, mainly concentrated in mitochondrial ATP-sensitive potassium channel, NF-kappa B molecular mechanism of signal transduction, and protein kinase C; mitochondrial mechanism is one of the important links. It has been demonstrated that a variety of signaling pathways and neural mediators are involved in the mechanisms of remote ischemic preconditioning, such as opioids, nitric oxide, adenosine, tumor necrosis factor *α*, and prostaglandins. However, it is still not clear how the signaling pathway is adapted to the oxygen free radical and calcium overload in the mitochondria.

Advanced glycosylation end-products receptor (RAGE) combined with a variety of ligands can promote intracellular oxidative stress generation and further activate the mitogen activated protein kinase (MAPK) family, NF-kappa B, and promote the release of inflammatory cytokines. RAGE plays an important role in myocardial ischemia-reperfusion injury. Previous studies had found that RAGE-ligand signaling pathway is associated with myocardial cell mitochondrial dysfunction in the process of ischemia-reperfusion (IR) injury [9, 10]. RIPostC has been shown to improve mitochondrial function and reduce inflammation reactions to reduce myocardial reperfusion injury. There were no reports of whether RAGE-ligand signaling pathways are involved in RIPostC to reduce myocardial ischemia-reperfusion injury.

In this study, we established murine model of myocardial ischemia-reperfusion injury to observe the effect of RAGE-HMGB1 on myocardial injury, protein expression of myocardial tissue, and cardiac function of ischemic. We investigated the hypothesis that RIPostC confers protection against IR injury by downregulating expression of RAGE in ischemic myocardium. We have also found that RIPostC limit myocardial infarct size and improve cardiac contractility through the inhibition of RAGE-HMGB1 and activation of RISK pathways.

## 2. Materials and Methods

### 2.1. Animals

In this study, C57BL/6 male mice (SPF grade, aged 8–12 weeks, weighing 20–25 g) were obtained from the Experimental Animal Center of Nanjing Medical University. Before the experiments were carried out, the mice were provided with 1 week of adaptive feeding and they were given a lot of water. The lab room was kept silent, the humidity was 55–70%, temperature was 23 ± 1°C, and fluorescent lighting was 12 h bright/12 h dark. We put every five mice in each cage covered with wood chips, and the animals were fasted for 12 hours before the experiment. In order to facilitate the observation and recording, each mouse was marked with a number tag on the ear. The study was in accordance with the standard of laboratory animal care and use, and the animal protocol was approved by the Ethics Review of Lab Animal Use Application of Nanjing Medical University (IACUC-1702003) and according to the Guide for the Care and Use of Laboratory Animals published by the US National Institutes of Health (NIH Publication number 85-23, revised 1996).

### 2.2. Ischemia/Reperfusion Model

Firstly, the mice were anesthetized with 1% sodium pentobarbital (50 mg/kg) fixed in the supine position, and the skin of neck and chest was disinfected. Then trachea incubation was performed and mechanical ventilation was established (respiratory rate of 150 times/min, suction ratio of 2 : 1, and tidal volume of 3 ml). Left thoracotomy was performed to expose the heart. Secondly, under microscope, we put 8-0 ophthalmic suture across the surface of myocardium below the left atrial appendage. A polyethylene hose about 2 mm in diameter was placed between the myocardial tissue and suture in a knot. After ligation of the left coronary artery, we could observe the color of the left anterior wall of the myocardium from red to white, and wall motion was significantly weakened or disappeared. Reperfusion was accomplished by loosening the knot, and we can confirm it by return of red color of the region. The mice of I/R group or RIPostC group suffered 45 min of ischemia and 120 min of reperfusion (I-45/R-120) with or without RIPostC. The hearts of mice in sham group were also exposed but without tying the suture on the LAD. Lastly, the thorax was closed. After mice restored spontaneous breathing, we pulled out the endotracheal intubation and placed them on the table waiting for their revival. The heart rate, electrocardiogram, and respiratory rate were continuously monitored in the whole process.

### 2.3. Remote Ischemic Postconditioning Protocol

The mice were anesthetized by intraperitoneal injection of 1% pentobarbital sodium, and the left lower limb inguinal was disinfected. The left femoral artery was exposed by being separated from the femoral vein and nerve. After the I/R model was established, limb RIPostC was performed using clamps immediately after the beginning of myocardial reperfusion, by three cycles of 5-minute occlusion and 5-minute release of the unilateral femoral artery. Ischemia was confirmed by pallor of the paw. Reperfusion was followed by clip removal and confirmed by return of blood supply and restoration of normal color.

### 2.4. Experimental Protocol

**Figure 1:**
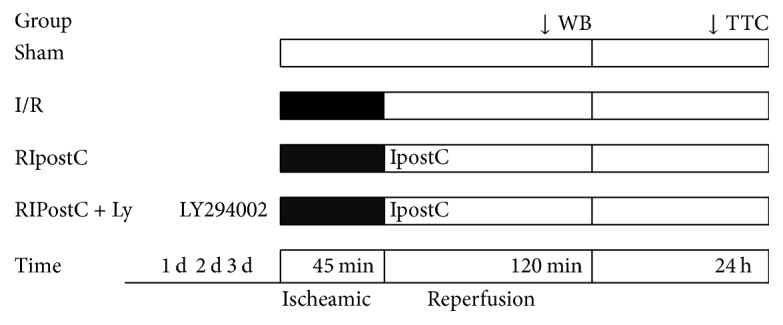
Experimental protocols. Mice were randomly assigned into five groups: (a) sham group (mice underwent sham surgery); (b) I/R group (mice underwent 40 min left anterior descending artery occlusion, followed by 120 min reperfusion); (c) RIPostC group (mice were underwent ischemic reperfusion injury, where remote conditioning was applied at the beginning of cardiac reperfusion). (d) RIPostC + Ly group (mice were injected with the PI3K inhibitor LY294002 and then underwent ischemic reperfusion injury, where remote conditioning was applied at the beginning of cardiac reperfusion). I/R: ischemia/reperfusion; RIPostC: remote ischemic postconditioning.

The mice were randomly divided into four groups subjected to different protocols (Figure 1): (1) sham group (*n* = 10; mice underwent thoracotomy but no LAD occlusion); (2) I/R group (*n* = 10; mice were subjected to left anterior descending artery occlusion for 45 min, followed by 120 min reperfusion); (3) RIPostC group (*n* = 22; mice underwent the same surgery as I/R group; additionally, at the beginning of coronary reperfusion, the mice were subjected to 3 cycles of 5-minute unilateral limb occlusion and 5-minute reperfusion); and (4) RIPostC + Ly group. To examine the role of PI3K/Akt signaling in cardioprotection of RIPostC, wild-type mice were injected with the PI3K inhibitor LY294002 (40 mg/kg i.p, dissolved in 0.5% DMSO, MedChemExpress, USA). In addition, 12 mice from the RIPostC group were sacrificed at 24 hours, 3 days, 5 days, and 7 days after reperfusion, and the myocardial tissue from infarction area was obtained for Western blot analysis.

### 2.5. Assessment of Myocardial Infarct Size

Infarct size was established by Evans blue and TTC staining as described previously [11]. Following 24-hour reperfusion, myocardial infarct size was assessed to measure the extent of IR injury (*n* = 5). After myocardial ischemia and reperfusion, LAD was reoccluded in the original position, and 2 ml of 0.5% Evans blue dye was slowly injected into the abdominal aorta. After the heart was blue stained, it was frozen in −20°C for 20 min and cut off. The heart was sliced into 4-5 sections, each about 1-2 mm thick, serially along longitudinal axis of left ventricular. The normal heart tissue could be identified as blue and the ischemia area as pink. Then, the sections were stained in 1.5% triphenyltetrazolium chloride (TTC, Biosharp, USA) for 30 min at 37°C and then fixed in the 4% paraformaldehyde for 2 hours. The area of necrosis (AN) appeared as grey white, while the noninfarct but at risk area was red. The mean area of nonischemic, ischemic (AAR), and infarct area (AN) was determined by HPIAS-2000 computer image analysis system.

### 2.6. Echocardiography Measurement of Cardiac Function

At the end of 24-hour reperfusion, cardiac function was evaluated by using a high-frequency ultrasound system Vevo770 (Visual Sonics Inc., Canada) with a 35 MHz central frequency scan head. Mice (*n* = 5, each group) were anesthetized with pentobarbital sodium (50 mg/kg) and positioned on the table. Two-dimensional image was obtained by using the conductive adhesive to connect the mouse limbs to the four electrodes and fixed with the adhesive tape. The scan head was placed on the left chest of the mice and the two-dimensional image was obtained at the short axis level. M-type echocardiography was obtained at the level of the papillary axis perpendicular to the interventricular septum and the posterior wall of the left ventricle. The scanning velocity was 800 mm/s. We measured the left ventricular end-systolic diameter (LVESd), left ventricular end-diastolic diameter (LVEDd), left ventricular posterior wall end-systolic thickness (LVPW), interventricular septum end-systolic thickness (IVSs), and interventricular septum end-diastolic thickness (IVSd). The left ventricular ejection fraction (LVEF%), left ventricular fractional shortening (LVFS%), and stroke volume (SV) were calculated by Visual sonics Vevo770 software. Each of the mice underwent an average of 3–5 consecutive cardiac cycles of ultrasound process.

### 2.7. Western Blot Analysis

For direct detection of protein levels, the heart was quickly cut off after 120 min of reperfusion; total protein was obtained from the heart tissue according to the manufacturer's instructions. Protein concentration was determined by BCA Protein Assay Kit (KeyGen BioTECH, China). 30 ug of total proteins was separated by electrophoresis on SDS-PAGE and then transferred onto 0.45 *μ*m PVDF membranes. The membranes were blocked for 2 h in 5% skim milk at room temperature and incubated with primary antibodies at 4°C overnight. Immunoblots were performed using the following antibodies: RAGE (Cell Signaling Technology, USA), HMGB1 (Cell Signaling Technology, USA), phosphorylated ERK1/2 (Cell Signaling Technology, USA), total ERK (Jiancheng Technology, CHINA), phosphorylated Akt (Cell Signaling Technology, USA), total Akt (Jiancheng Technology, CHINA), and rabbit anti-GAPDH (Bioworld, China). Then the membranes were washed with TBST. We blocked the protein with phosphate-horseradish peroxidase-conjugated secondary antibody (Cell Signaling Technology, USA) at room temperature for 2 hours. The complexes were detected by using ECL kit (Thermo, USA). The images were analyzed with digital gel imaging system (Bio-Rad, USA).

### 2.8. Statistical Analysis

We performed statistical analysis by SPSS software, version 19.0. All count data are expressed as mean ± standard deviation (*X* ± SD), and the measurement data is expressed as a percentage (%). The difference between the two groups was statistically analyzed using a separate sample *t*-test; the comparison among different groups was compared with one-way ANOVA with post hoc test. The variance homogeneity test of the data is carried out using the normality test and the Levene test. *P* value < 0.05 was considered to indicate a statistically significant difference.

## 3. Results

### 3.1. Cardiac Function

**Figure 2:**
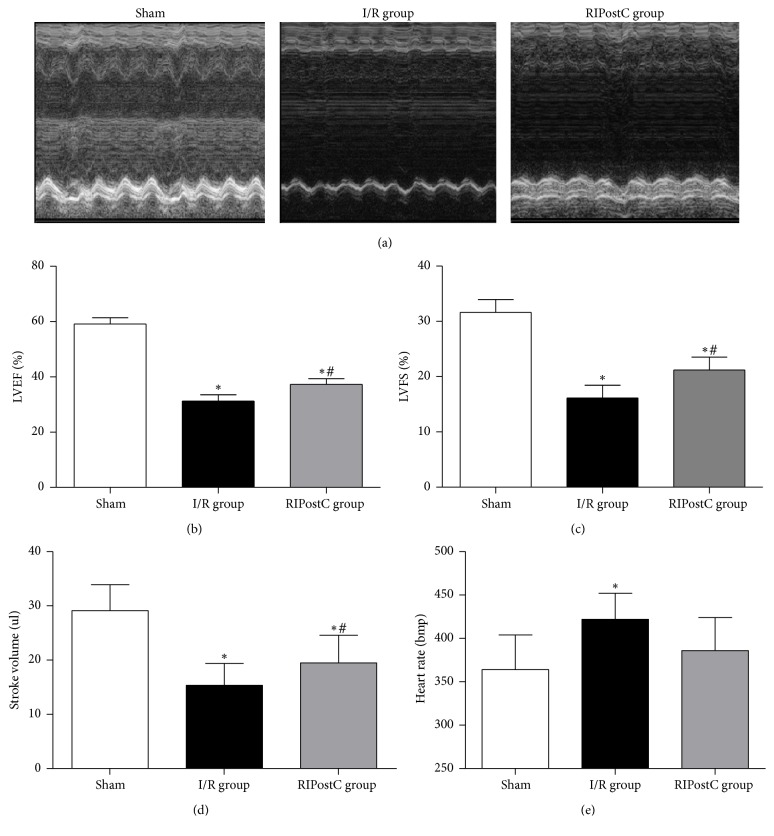
RIPostC in the effects of cardiac function in I/R mice. (a) Representative M-mode echocardiographic images of mice. (b) Analysis of left ventricular ejection fraction (LVEF) at 24 hours after I/R. (c) Analysis of left ventricular fractional shortening (LVFS) at 24 hours after I/R. (d) Analysis of left stroke volume at 24 hours after I/R. (e) Analysis of heart rate at 24 hours after I/R.* n* = 5 in the sham group,* n* = 5 in the I/R groups, and* n *= 5 in the RIPsotC group. ^*∗*^*P* < 0.05 versus sham group, ^#^*P* < 0.05 versus I/R group.

LVEF and LVFS of mice in I/R group were significantly decreased compared with control group (LVEF: 59.07 ± 2.24% versus 31.25 ± 2.27%, *P* < 0.01; LVFS: 31.60 ± 2.35% versus 16.16 ± 2.28%, *P* < 0.01). Echocardiography of RIPostC group showed a significant increase in LVEF (37.25 ± 2.06% versus 31.25 ± 2.27%, *P* < 0.05) and LVFS (21.22 ± 2.34% versus 16.16 ± 2.28%, *P* < 0.05) compared with the I/R group (Figures 2(a)–2(c)). Stroke volume (SV) of I/R group was significantly decreased compared with control group (15.36 ± 4.01 ul versus 29.10 ± 4.81 ul, *P* < 0.05), while RIPostC could increase SV compared to the mice of I/R group. The heart rate of I/R group was significantly higher than that of control group (364 ± 40 bmp versus 422 ± 30 bmp, *P* < 0.05). The heart rate of RIPostC group (386 ± 38 bmp) was lower than that of I/R group (422 ± 30 bmp), but there was no significant difference between the two groups (*P* > 0.05) (Figures 2(d)-2(e)).

### 3.2. Infarct Size

**Figure 3:**
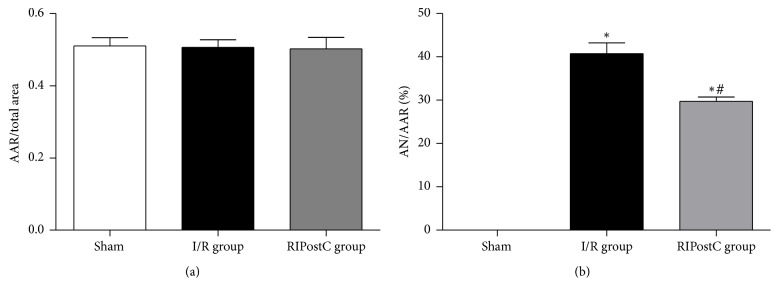
Myocardial infarct size 24 h after reperfusion. (a) AAR/total area of each group. Area at risk as percentage of the area of total left ventricle did not differ between the two groups. (b) Infarct size of each group. Infarct size expressed as percentage infarction of the area at risk in hearts. Data are expressed as the mean ± standard deviation. AN, area of necrosis; AAR, area at risk; IR, ischemia/reperfusion; RIPostC, remote ischemic postconditioning. ^*∗*^*P* < 0.05 versus sham group; ^#^*P* < 0.05 versus I/R group.

There was no significant difference in the risk areas of the total LV (AAR) among RIPostC group, control group, and I/R group (0.51 ± 0.23 versus 0.50 ± 0.21 versus 0.51 ± 0.15, *P* > 0.05). Compared with the I/R group, the infarct area (AN/AAR) was significantly reduced in the RIPostC group (29.75 ± 1.97% versus 40.72 ± 2.49%, *P* < 0.01). The weights of mice were similar among three groups (23.70 ± 1.04 g versus 23.68 ± 0.92 g versus 23.43 ± 0.69 g, *P* > 0.05) (Figures 3(a)–3(b)).

### 3.3. Western Blot of HMGB1-RAGE Pathways

**Figure 4:**
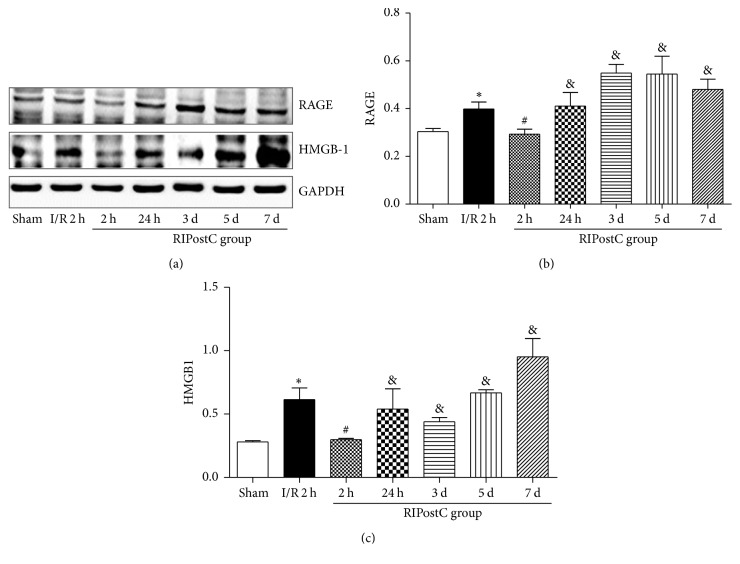
The expression levels of RAGE and HMGB1 in heart tissue. (a) Western blot analysis of RAGE and HMGB1. GAPDH was used as an internal control. (b) Protein levels of RAGE under different treatments before reperfusion in heart. (c) Protein levels of HMGB1 between I/R and RIPostC group. ^*∗*^*P* < 0.05 versus sham group, ^#^*P* < 0.05 versus I/R group, and ^&^*P* < 0.05 versus RIPostC at 2 h.

The RAGE levels were significantly increased in the I/R group compared to the sham group (0.40 ± 0.029 versus 0.30 ± 0.012, *P* < 0.05). In the RIPostC group, the levels of RAGE were lower values than in I/R group following 120 min of reperfusion (0.29 ± 0.021 versus 0.40 ± 0.029, *P* < 0.05). Similarly, the levels of HMGB1 in the I/R group were significantly higher than those in the sham group (0.61 ± 0.093 versus 0.30 ± 0.010, *P* < 0.01). Compared with the I/R group, RIPostC could decrease the expression of HMGB1 following 120 min of reperfusion (0.30 ± 0.010 versus 0.61 ± 0.093, *P* < 0.05). In RIPostC group, the expressions of RAGE and HMGB1 in myocardial tissue were increased again at 24 hours, 3 days, 5 days, and 7 days following reperfusion than these at 120 min of reperfusion (Figures 4(a)–4(c)).

### 3.4. Western Blot of Reperfusion Injury Salvage Kinase (RISK) Pathways

**Figure 5:**
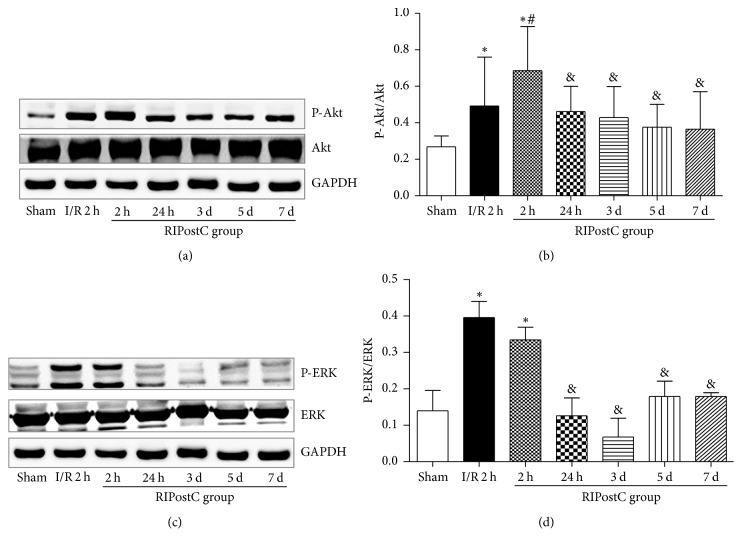
Effect of RIPostC on reperfusion injury salvage kinase (Akt, ERK1/2) pathway. (a) Western blot analysis of total and phosphorylated Akt and ERK1/2. GAPDH was used as an internal control. (b) Densitometry for P-AKT expression normalized to GAPDH at different time after I/R. (c) Densitometry for P-ERK1/2 expression normalized to GAPDH at different time after I/R. ^*∗*^*P* < 0.05 versus sham group, ^#^*P* < 0.05 versus I/R group, and ^&^*P* < 0.05 versus RIPostC at 2 h.

In the I/R group, the phosphorylation levels of Akt were significantly increased following 120 min of reperfusion, compared with the sham group (0.49 ± 0.027 versus 0.27 ± 0.059, *P* < 0.05). In the RIPostC group, further increases in the phosphorylation levels of Akt were detected following 120 min of reperfusion (0.69 ± 0.024 versus 0.49 ± 0.027, *P* < 0.05). However, LY294002 significantly attenuated RIPostC-increased levels of Akt phosphorylation following 120 min of reperfusion (0.56 ± 0.046 versus 0.81 ± 0.058, *P* < 0.05, shown in Figure supplement 1). In the RIPostC group, compared with 120 min of reperfusion, the phosphorylation levels of Akt were significantly decreased at 24 hours, the third day, fifth day, and seventh day of reperfusion (Figures 5(a)-5(b)).

The levels of p-ERK1/2 significantly increased following 120 min of reperfusion in the I/R group and RIPostC group, compared with the sham group (0.40 ± 0.044, 0.33 ± 0.035 versus 0.14 ± 0.055, *P* < 0.05). However, no significant differences were observed between the I/R group and RIPostC group (*P* < 0.05) (Figures 5(c)-5(d)).

## 4. Discussion

In this study, we demonstrated that (1) RIPostC could provide significant protection against myocardial ischemia-reperfusion injury, as evidenced by reduced infarct size and improvement of cardiac function. (2) RAGE-HMGB1 signaling pathway plays an important role in MIRI. (3) RIPostC can attenuate the injury of ischemia-reperfusion myocardium by inhibition of RAGE-HMGB1 signaling pathway and activation of RISK signaling pathways.

Myocardial ischemia-reperfusion injury (MIRI) is a common pathophysiological process, a serious threat to the health of patients. When the ischemic myocardium is reperfused, some harmful effects on clinical outcome are also accompanied, including myocardial dysfunction, ventricular arrhythmias, abnormal metabolism, microvascular dysfunction, and the changes of myocardial ultrastructure, which can lead to cell dysfunction and death [3]. How to reduce the damage caused by ischemia reperfusion has become the key to the treatment of ischemic myocardial diseases.

Remote ischemic conditioning (RIC), induced by several episodes of brief ischemia and reperfusion at a distance organ, has been developed as an effective strategy against the harmful effects of acute myocardial ischemia-reperfusion injury [12]. In comparison with conventional local ischemic conditioning, the remote ischemic conditioning can be performed noninvasively using a blood pressure cuff on the upper/lower limb making it more clinically feasible [13]. Unlike local ischemic conditioning, a promising treatment strategy for IRI in patients is RIC. In different animal investigations [5, 6] and randomized clinical trials RIPostC have been reported to be beneficial [7, 8]. However, the mechanism of cardioprotective effect induced by remote ischemic postconditioning remains poorly understood. At present, the mechanisms of RIPostC are mainly focused on anti-inflammatory, antioxidant, and antiapoptosis.

This study showed that remote ischemic postconditioning can effectively reduce the area of myocardial infarction. The infarct area (AN/AAR) of mice was 40.72 ± 2.49% in the I/R group and 29.75 ± 0.97% in the RIPostC group. These results suggest that the remote ischemic postconditioning can effectively alleviate myocardial ischemia-reperfusion injury. Kerendi et al. first found that remote ischemic postconditioning can reduce myocardial infarction area by 50% in 2005 [14]. In this animal experiment, the area of myocardial infarction was 49 ± 4% in ischemia-reperfusion group and 25 ± 4% in remote ischemic postconditioning group. In 2007, Andreka et al. [15] first verified the effect of RIPostC on reducing myocardial infarction in the pig ischemia-reperfusion model. They found that RIPostC can make the myocardial infarction area decrease by 22%. In this study, myocardial infarct size was reduced by 26.94% in RIPostC group compared with that in I/R group, which was consistent with the previous studies.

In this experiment, we evaluated the cardiac function by echocardiography measurement 24 hours after reperfusion. RIPostC group showed a significant increase in LVEF and LVFS of myocardial ischemia-reperfusion mice. These indicated that remote ischemic postconditioning can further improve the cardiac function in mice undergoing reperfusion injury.

Inflammatory response is one of important mechanisms for myocardial ischemia-reperfusion injury [16]. It has been demonstrated that MIRI can activate nuclear factor-kappa B (NF-*κ*B) signaling pathway at a very early stage of reperfusion [17], which further led to the release of serum proinflammatory cytokines, such as TNF-*α* and IL-6 [18]. Also, the TNF-*α* released in the process of myocardial IRI can induce cascade of proinflammatory cytokines, which further increases the formation of proinflammatory cytokines such as IL-1 and IL-6. Subsequently, increased serum proinflammatory cytokines result in Ca^2+^ dyshomeostasis in the cardiocytes, decreasing the myocardial contractile function [19]. Activation of NF-*κ*B in the ischemic myocardium can induce mitochondria damage evidenced by mitochondria swelling, disruption of crista, and reduced density, which would lead to irreversible myocardial ischemia-reperfusion injury [20].

Advanced glycosylation end-products receptor (RAGE) is a member of the immunoglobulin superfamily [21, 22]. RAGE is expressed on multiple cell types, such as cardiomyocytes, microglia, glomerular epithelial cells, and alveolar epithelial cells; for example, see [23–25]. RAGE combined with a variety of ligands can promote intracellular oxidative stress generation, further activate the mitogen activated protein kinase (MAPK) family and NF-kappa B, and promote the release of inflammatory cytokines. Previous reports show that RAGE signaling plays an important role in ischemia-reperfusion injury in various tissues and organs such as heart, liver, and brain [26–29]. RAGE may mediate oxidative stress and the release of inflammatory cytokines, by combination with its ligands [30, 31]. A recent study confirmed that HMGB1 can function as a mediator to modulate inflammatory response and cell injury during the early stage of myocardial IRI as well as the classic early proinflammatory cytokines. Moreover, HMGB1 is able to promote the release of TNF-*α* and IL-6 and consequently deteriorated the myocardial IRI [32, 33]. Liu et al. demonstrated the alternative cross-talk between RAGE overexpression and nitrative Trx inactivation, suggesting that interventions interfering with their interaction may be novel means of mitigating diabetic MI/R injury [9]. Increased myocardial injury is a result of the activation when HMGB1 binds to RAGE, proinflammatory pathways.

Up to now, there has been no study evaluating the effects of RIPostC on RAGE-HMGB1 signaling pathway during the process of myocardial ischemia-reperfusion injury. Our study found that the protein levels of RAGE and HMGB1 were significantly increased in the I/R group compared to these in the sham group, while RIPostC can decrease the protein levels of RAGE and HMGB1 following 120 min of reperfusion. This study showed that HMGB1-RAGE signaling pathway plays an important role in myocardial ischemia-reperfusion injury. Our study first confirmed that RIPostC can significantly inhibit myocardial local inflammatory responses by downregulation of RAGE/HMGB1 signaling pathways to alleviate myocardial ischemia-reperfusion injury. But there is another possibility where RIPostC reduced myocardial inflammatory response during ischemia-reperfusion, decreasing the infiltration of neutrophils and macrophages. We cannot rule out that the reduced expression of HMGB1 and RAGE at 2 h after reperfusion might be the result of cardioprotection by RIPostC.

Previous studies have demonstrated that the RISK and SAFE pathways play an important role in remote ischemic conditioning. RIPC can reduce infraction areas by activation of PI3K and its downstream targets Akt and glycogen synthase kinase 3*β* (GSK 3*β*) [34, 35]; simultaneously it can activate ERK pathway trigger adenosine and subsequent transactivation of the epidermal growth factor receptor [36]. Hausenloy et al. demonstrated that RISK activation present biphasicity during the preconditioning cycles and again at early reperfusion [37]. The present study demonstrated that RIPostC also could increase the phosphorylation of Akt and ERK1/2, suggesting that the protective effects of RIPostC are associated with activation of the RISK pathways, being consistent with previous studies [38, 39].

The present study indicated that RIPostC can activate PI3K-Akt signaling pathway and further inhibit oxidative stress, having antiapoptosis effect, to achieve myocardial protection effect. However, the phosphorylation of ERK had no significant difference between the I/R group and the RIPostC group. The role of ERK1/2 signaling pathway in the protective effect of RIPostC was not observed. Previous experiments of remote postconditioning on ERK1/2 signaling pathway were not consistent, the role and mechanism of ERK in ischemic reperfusion and RIC are not clear, and further research is needed. Furthermore, we used PI3K inhibitor ly294002 to observe the effect of RIPostC on PI3K-Akt signaling pathway. The results showed that RIPostC increased Akt expression 2 hours after reperfusion, and PI3K inhibited the increase of Akt under the action of RIPostC. These results indicated that the role of PI3K signaling pathway in RIPostC is very important.

In our present study on remote ischemic postconditioning, we assessed the time courses of the protein expression of HMGB1-RAGE and phosphorylation of Akt, ERK1/2 by the RIPostC maneuver in the myocardium of mice at different time after reperfusion. The levels of HMGB1 and RAGE were higher at 24 hours of reperfusion than at 120 min of reperfusion, and the expression of P-Akt was gradually decreased at 24 hours after myocardial ischemia reperfusion. This indicated that the protective effect of RIPostC had a time limitation. The activation of PI3K/Akt signal transduction was mainly focused on the early stage of remote ischemic postconditioning, while this protective effect would gradually dissipate in the later stage of reperfusion (>24 h). Thus, RIPostC plays an important role in inhibiting the inflammatory reaction and activating the RISK pathway in the early stage of reperfusion.

In conclusion, the present study demonstrated that RIPostC was effective in protecting against myocardial ischemia-reperfusion injury. The cardioprotective effects of RIPostC were associated with inhibition of RAGE-HMGB1 and increased activation of PI3K/Akt signaling pathway, but this effect has certain timeliness.

## Supplementary Materials

Supplementary Materials Figure supplement 1: effect of PI3K inhibitor on reperfusion injury salvage kinase pathway. (a) Western blot analysis of total and phosphorylated Akt. GAPDH was used as an internal control. (b) Densitometry for P-Akt expression normalized to GAPDH at different time after I/R.
