# Supplementary material for: Remote Ischemic Postconditioning Protects against Myocardial Ischemia-Reperfusion Injury by Inhibition of the RAGE-HMGB1 Pathway
Source: Biomed Res Int. 2018 Jan 23;2018:4565630. doi: 10.1155/2018/4565630 (PMC5896327; doi:10.1155/2018/4565630)
Supplement: Supplementary Materials — Figure supplement 1: effect of PI3K inhibitor on reperfusion injury salvage kinase pathway. (a) Western blot analysis of total and phosphorylated Akt. GAPDH was used as an internal control. (b) Densitometry for P-Akt expression normalized to GAPDH at different time after I/R. [file 4565630.f1.docx]

**Figure supplement1. Effect of PI3K inhibitor on Reperfusion injury salvage kinase pathway.** (a)Western blot analysis of total and phosphorylated Akt. GAPDH was used as an internal control. (b) Densitometry for P-AKT expression normalized to GAPDH at different time after 1/R. Data are expressed as the mean ± standard deviation. *P<0.05 versus sham group, **^#^**P<0.05 versus I/R group, ^&^P＜0.05 versus RIPostC group.
